# Automatic water irrigation synchronized with the electrosurgical unit: Bubble-free underwater endoscopic submucosal dissection

**DOI:** 10.1055/a-2316-9305

**Published:** 2024-06-05

**Authors:** Motoki Sasaki, Teppei Masunaga, Kurato Miyazaki, Kiyokazu Nakajima, Naohisa Yahagi, Motohiko Kato

**Affiliations:** 1 Division of Research and Development for Minimally Invasive Treatment, Cancer Center, Keio University School of Medicine, Tokyo, Japan; 2 Department of Next Generation Endoscopic Intervention (Project ENGINE), Osaka University Graduate School of Medicine, Suita, Japan; 3 Division of Gastroenterology and Hepatology, Department of Internal Medicine, Keio University School of Medicine, Tokyo, Japan; 4 Center for Diagnostic and Therapeutic Endoscopy, Keio University School of Medicine, Tokyo, Japan

The water pressure method is a underwater ESD technique, which facilitates direct visualization of the submucosa using a water stream through the waterjet channel of an endoscope 1 . This method significantly improves endoscopic exposure in difficult conditions such as the initiation of the submucosal dissection, dissection in a narrow space, and dissection of fibrotic tissue, and has been reported as useful for technically challenging ESD 2 3 4 . However, there is a problem in that the heat generated by the activation of the electrosurgical device at the lesion site creates bubbles in the water that often obstruct endoscopic visualization. In addition, especially when a tapered hood attachment is used, those bubbles are often trapped inside the attachment and are difficult to remove.

To solve this drawback, we have developed a modified underwater ESD technique that uses a water irrigation pump controlled synchronously with the activation from the electrosurgical unit. An endoscopic water irrigation pump (EIP2; Erbe, Tübingen, Germany) ( Fig. 1 ) is connected to the waterjet channel by a dedicated tube. This EIP2 pump also has a wired connection to the VIO3 electrosurgical unit (Erbe), that enables simultaneous activation of the pump and the VIO3 ( Fig. 2 , Fig. 3 ). Water irrigation is done automatically when incision or coagulation currents are activated, thereby removing any generated bubbles before they can be trapped in the hood attachment and enabling a continuously clear view for ESD performance ( Fig. 4 , Video 1 ). We use the EIP2 with an output power of 50%–60%, but we recommend adjusting the power on a case-by case basis.

**Fig. 1:**
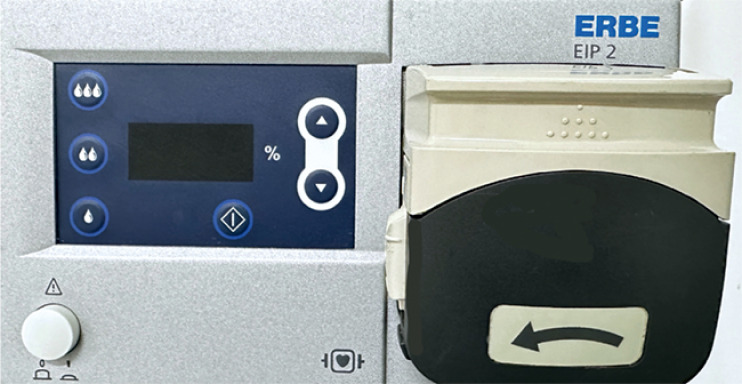
Water irrigation pump (EIP2; Erbe, Tübingen, Germany).

**Fig. 2:**
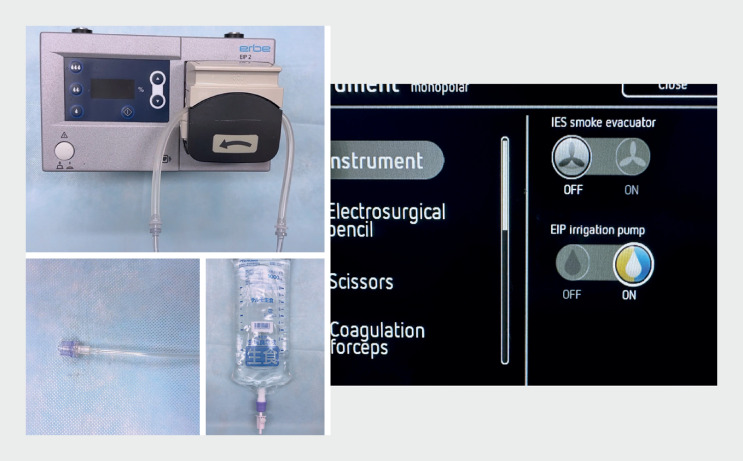
Preparation of the EIP2 water irrigation pump and setting of the VIO3 electrosurgical unit.

**Fig. 3:**
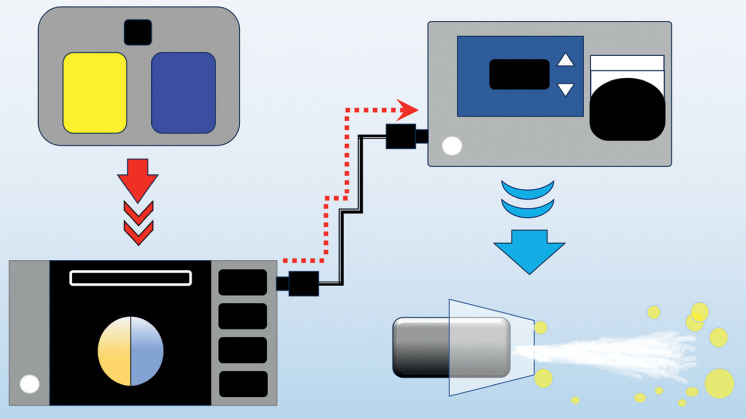
Schematic of the automatic and synchronized water irrigation system.

**Fig. 4:**
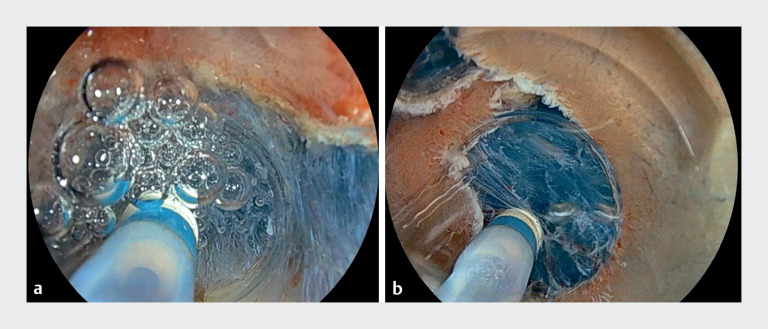
Comparison of endoscopic image of bubbles inside the endoscopic tip attachment after 1 s of instrument activation. **a** Conventional underwater endoscopic submucosal dissection (underwater ESD) using the water pressure method. **b** Using the automatic synchronized water irrigation system (bubble-free underwater ESD).

Bubble-free underwater endoscopic submucosal dissection (underwater ESD) using an automatic water irrigation system that synchronizes with an electrosurgical unit.Video 1

The bubbles generated during underwater ESD compromise endoscopic visibility, which may lead to loss of procedural precision. Our new technique, named “bubble-free underwater ESD,” can be a simple and practical solution.

Endoscopy_UCTN_Code_TTT_1AQ_2AD

## Notes

Endoscopy E-Videos https://eref.thieme.de/e-videos
*E-Videos* is an open access online section of the journal *Endoscopy* , reporting on interesting cases and new techniques in gastroenterological endoscopy. All papers include a high-quality video and are published with a Creative Commons CC-BY license. Endoscopy E-Videos qualify for HINARI discounts and waivers and eligibility is automatically checked during the submission process. We grant 100% waivers to articles whose corresponding authors are based in Group A countries and 50% waivers to those who are based in Group B countries as classified by Research4Life (see: https://www.research4life.org/access/eligibility/ ). This section has its own submission website at https://mc.manuscriptcentral.com/e-videos .
